# A Study on the Association between Low Maternal Serum Magnesium Level and Preterm Labour

**DOI:** 10.1155/2014/704875

**Published:** 2014-04-13

**Authors:** Kehinde S. Okunade, Ayodeji A. Oluwole, Maymunah A. Adegbesan-Omilabu

**Affiliations:** Department of Obstetrics & Gynaecology, Lagos University Teaching Hospital, Lagos, Nigeria

## Abstract

*Objectives.* The study was aimed to assess the association between low maternal serum magnesium levels and preterm labour.* Methods.* It is a cross-sectional case-control study in which eligible participants were pregnant women admitted in labour within the labour ward complex of a Lagos tertiary hospital. Relevant data were extracted from the case records of these women and blood samples were obtained from all participants and serum magnesium levels measured. * Results.* The study showed that 36% of the study patients had varying degrees of hypomagnesaemia. The relative risk indicates that preterm labour is 1.83 times higher among the patients with low serum magnesium (less than 1.6 mg/dL). The mean difference in serum magnesium levels in both groups was statistically significant (*P<*0.05). * Conclusion.* We can infer that low serum magnesium (hypomagnesaemia) is associated with preterm onset of labour. We can, also from this finding, formulate a proposition that would help in preventing preterm labour and birth with the use of prophylactic oral magnesium supplementation among patients with higher risk for development of preterm labour.

## 1. Introduction

Prematurity represents a significant obstetric concern and has become more common in recent years. Since 1981, the rate of preterm birth has increased by about 30% [1]. One out of every eight babies is now born prematurely [2, 3], although the incidence varies considerably with the population studied and the majority is preceded by spontaneous preterm labour.

Preterm delivery is defined as birth occurring prior to 37 completed weeks of gestation [4] while preterm labour is defined as labour that occurs with regular and frequent uterine contractions causing progressive cervical changes before 37 completed weeks of gestation. It accounts for 10–15% of all pregnancies [5]. The incidence also varies with population studied.

Preterm delivery is not only a leading cause of neonatal morbidity and mortality [3, 6]; its long-term sequelae pose a serious problem for the offspring and for the mother [7]. The exact cause of preterm labour and delivery remains elusive and is likely to be multifactorial; in 50% of cases, it is spontaneous and idiopathic, although several potential risk factors have been identified. The main one among them is premature rupture of membrane (PROM), and others are multiple pregnancy, polyhydramnios, hypertensive disorders of pregnancy, infections, cervical incompetence, antepartum haemorrhage, fetal and uterine anomalies, anaemia, heavy work, smoking, and so forth [8, 9]. It is also related to socioeconomic status and geographic location [8–10].

Prevention of viable spontaneous preterm birth through screening is one of the key aims of antenatal care as these have implications for child, mother, and society. If women can be identified to be at high risk in early pregnancy, they can be targeted for more intensive antenatal surveillance and prophylactic interventions (primary prevention). However, the disease mechanisms behind these problems are not well understood. Consequently, tests for their prediction and treatments for their prevention are not well developed. Clinically, it would be useful to be able to predict who will deliver preterm. The predictors may be used in the management of women at high risk for preterm labour such as women with previous preterm labour and also can be used as a part of a management protocol to individualize patient care [11, 12]. Several markers more directly related to preterm labour have recently been proposed, some of which relate to direct causes of preterm labour such as cervical ultrasound measurement, fetal fibronectin (FFN), salivary estriol, serum CRH, and bacterial vaginosis [13]. Several of these have predictive values, which are potentially useful for clinical practice but are at present still subjects of research interests [13] and thus none are routinely used in Nigeria.

Besides varied aetiology, preterm labour may be due to alteration in basic biochemical functions of the body at cellular level [14]. This supports the need to investigate the association between trace elements and preterm labour. In this regard, magnesium, one of the trace elements, has become the subject of interest [15]. Hypomagnesaemia during pregnancy decreases the magnesium level in myometrium and a low magnesium concentration in pregnant human myometrium could be a cause of preterm labour [16–18]. Rising serum magnesium level serves to relax the uterine smooth muscle, thereby providing the basis for the previous use of magnesium sulphate as a tocolytic agent in pregnancy in North America [14, 19], although a Cochrane systematic review has now concluded that it is ineffective as a tocolytic agent and may even be harmful to the unborn baby [20].

However, due to the paucity of data in this regard in Nigeria and other black African countries, our study will therefore aim to investigate whether low maternal serum magnesium during pregnancy may be associated with preterm labour and delivery in the Nigerian black pregnant population and thereafter describe a generic framework for combining this screening information with designing a prophylactic intervention.

## 2. Materials and Methods

The study was conducted in the labour ward complex of a Lagos tertiary hospital in Nigeria. It was a cross sectional case-control study carried out over a period of 12 months among young, generally healthy pregnant women on admission while in labour.

The sample size (*N*) for each group in the study was determined using the statistical formula by Schlesselman [21]. A total number of 200 women were selected by consecutive sampling method for the study, having confirmed that they are in labour and they are grouped as follows:

 group-I (case): 100 cases with preterm labour of idiopathic aetiology occurring after 28 weeks (the earliest age of fetal viability in Nigeria) and before 37 completed weeks of gestation,
 group-II (control): 100 women with term labour occurring at and/or after 37 completed weeks of gestation.

Eligible participants were pregnant women aged 16 to 40 years (the usual age range of the Nigerian pregnant population) and have singleton gestation. Exclusion criteria included women with history of diabetes, HIV, significant intercurrent infections or other illnesses, multiple pregnancy, polyhydramnios, preeclampsia or other gestational disorders, current or previous history of smoking, and other described substance uses. Also excluded were patients with early pregnancy bleeding or conflicting data regarding gestational age. Medical records for the index pregnancy were sought for potentially eligible women and an informed written consent was obtained from each participant upon explanation of the nature and purpose of the study.

Relevant data were extracted from the case records of these women and a structured interviewer administered questionnaire was used for the data collection. Gestational duration was based upon gestation from participants' last normal menstrual period confirmed or modified by ultrasound. Social classes were determined using the Oyedeji socioeconomic classification scheme [22].

Venous blood samples were obtained by venipuncture and collected in lithium heparin specimen bottles. Magnesium level in serum was then analyzed using Xylidyl Blue Colorimetric Method [23]. If the labour pain stopped and pregnancy continued, the blood sample was discarded and the patient was excluded from the study.

The reference value for normal serum magnesium is 1.6–2.6 mg/dL [24]. Thus, low maternal serum magnesium pregnancies are defined as those in which maternal serum magnesium level was below 1.6 mg/dL. The coefficient of variation within and between assays of <5% was used.

All quantitative data were entered into a computer and analysed using SPSS version 17 for windows [25]. Descriptive statistics were then computed for all relevant data. The association of low maternal serum magnesium with preterm labour was tested using chi-square to determine the difference. All significances were reported at *P* < 0.05.

Ethical approval was obtained from the Hospital's Health Research and Ethics Committee prior to the commencement of the study and written consent was obtained from each participant before involvement in the study.

## 3. Results

Table 1 shows that the mean and standard deviation (mean ± SD) of age in the case and control groups were 29.75 ± 5.24 and 28.63 ± 5.92, respectively. There was no statistically significant difference between the two groups (*P* = 0.098). There were also no significant differences between the case and control groups' distributions regarding the parity (*P* = 0.534), marital status (*P* = 0.064), tribe (*P* = 0.830), religion (*P* = 0.106), and social class (*P* = 0.139).

**Figure 1:**
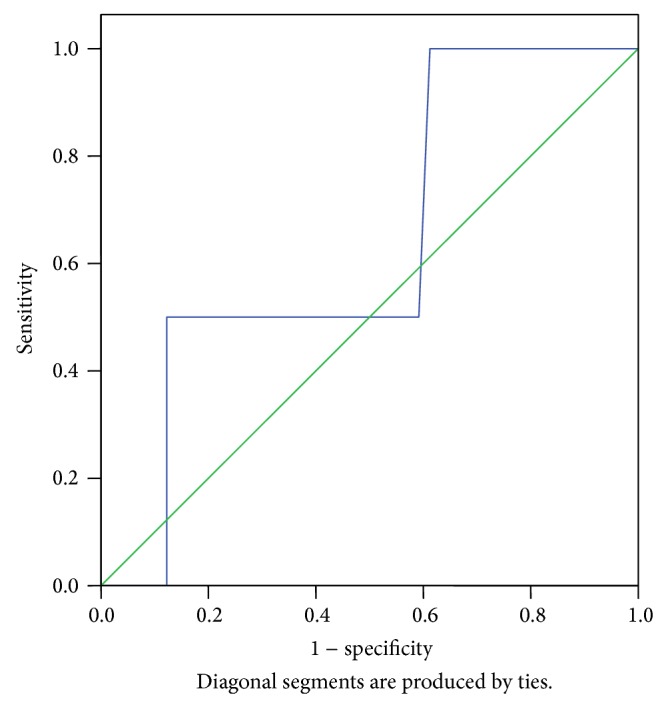
ROC curve for low maternal serum magnesium level (>1.6 mg/dL) and preterm labour.

In Table 2, 36% of the study patients have varying degrees of hypomagnesaemia. It was also revealed that 47% of the case patients had serum magnesium level less than 1.6 mg/dL, whereas only 25% of the control patients had this low serum magnesium level. The relative risk (RR) indicates that the risk of preterm labour is 1.83 times higher among the patients with low serum magnesium (less than 1.6 mg/dL). The mean difference in serum magnesium level in both groups was statistically significant (*P* = 0.024).  Table 3 shows the comparison of the present study with other previous studies on serum magnesium level in preterm labour. However, further analysis of the maternal serum magnesium cutoff value of less than 1.6 mg/dL as a predictive measure of preterm labour using the ROC curve (Figure 1) revealed that its sensitivity, specificity, positive, and negative predictive values were 50, 52, 73.8, and 60.6%, respectively.

It was revealed in Table 4 that there is no significant difference in the gestational age distribution of women who delivered preterm in the low and normal serum magnesium levels groups, respectively.  Table 5 also shows no statistically significant differences in the maternal age (*P* = 0.099) and social class (*P* = 0.101) of the study patients with low and normal serum magnesium levels, respectively. The mean serum magnesium level of the study patients was lower among mothers who had no previous history of abortion (1.80 ± 0.4 mg/dL) than those who had previous history of abortion (1.88 ± 0.4 mg/dL) but the mean difference is not statistically significant (RR-0.96, 95% CI-2.94 to 6.97; *P* = 0.716) whereas the mean serum magnesium level of mothers with previous history of preterm labour (1.66 ± 0.5 mg/dL) was lower than that of those who had no history of preterm labour (1.87 ± 0.4 mg/dL) and also with a mean difference that is not statistically significant (RR-1.14, 95% CI-1.01–5.50; *P* = 0.059).

## 4. Discussion

Several studies have examined the association between maternal serum magnesium levels and preterm labour but there is no available data in Nigeria and Africa to examine this relationship especially among the black pregnant population. Since we are trying to introduce a predictive test, all women within the usual age range of the Nigerian pregnant population (16–40 years) were included in our analyses.

Similar to the work of Shahid et al. [14], our study found no significant difference in age, parity, history of miscarriage, and socioeconomic status between the case and control groups which confirmed similarities in the two groups used in the study.

The incidence of hypomagnesaemia among the healthy parturients used in the study after excluding most of the major risk factors was found to be 36%. This is slightly lower than the incidence of 46% found among similar number of patients studied by Shahid et al. [14]; this difference may be explained by the cutoff points for magnesium (1.6 versus 1.9 mg/dL, resp.) used in the two studies.

The main focus of this study is the role of serum magnesium level in preterm labour and its relation with the aetiology of preterm labour. Past studies and reports have shown a decreased level of serum magnesium in preterm labour [14, 15, 26–28]. These were corroborated by our study where a reduction in mean serum magnesium level was found in cases of preterm labour (1.73 ± 0.4 versus 1.93 ± 0.4 mg/dL). This result is also found to be similar to and supported by other investigators. In a study carried out by Pushpo and Jagdish, the serum magnesium level in preterm labour was found to be 1.67 ± 0.23 mg/dL [27]. Shahid et al. found that the patients with preterm labour had significantly depressed serum magnesium level and the mean was 1.60 ± 0.466 and 1.87 ± 0.3 mg/dL, respectively [14, 26].

The finding from this study is also similar to the result obtained by Kamal et al. who found that the mean serum magnesium level in preterm labour cases was 1.4 mg/dL ± 0.22 SD and therefore suggested that the estimation of serum magnesium may prove to be a valuable tool in predicting the preterm onset of labour [15]. A study by Begum et al. also observed that there was significant reduction of serum magnesium (mean 1.77 ± 0.36) in women with preterm labour [28].

A relative risk of preterm labour was 1.83 among the patients with serum magnesium level less than 1.6 mg/dL compared to those who had a higher magnesium level and the difference was statistically significant (*P* = 0.024) which confirmed the study hypothesis. There are, however, other studies that found no relationship between maternal serum magnesium level and preterm labour [29–31]. These studies were supported by systematic reviews and other studies that revealed that the use of oral or parenteral magnesium had no effect on delaying the onset of preterm labour or prevention of premature birth [16, 29, 32, 33] and therefore it is no longer recommended for routine use as tocolytic [16]. However, reports from various studies [14, 26–30] showed significantly low serum magnesium levels in women with preterm labour indicating that hypomagnesaemia may in fact be a risk factor for preterm labour.

Our study also examined four important risk factors for preterm labour (maternal age, social class, previous history of abortion, and preterm labour). Although it was found by Cunningham et al. [34] in 2005 that there is a relationship between preterm labour and maternal ages and low socioeconomic status, such a finding was not obvious from our study (Table 6). This might be because sampling was obtained only from government hospitals in this present study and other similar studies and not in private hospitals like Cunningham's study, in which participants were almost entirely in similar upper socioeconomic groups. The variation in this finding to that of another study that showed low serum magnesium level in preterm labour cases may be because low serum magnesium level was found in patients belonging to the low socioeconomic status, thus relating the low level of magnesium from diet deficient in magnesium [35].

From this study also, history of previous preterm labour and midtrimester miscarriage which is both regarded as the most important predictors for preterm labour as indicated by past studies [14, 36] were not associated with corresponding reduction in serum magnesium levels. This is possible probably because of the varying biochemical changes that occur from one pregnancy to the other even in the same individual [15].

## 5. Limitations to the Study

Magnesium contamination may occur from use of glassware during the sample analysis and the use of haemolysed samples may produce falsely elevated results because of the high concentration of intracellular magnesium. Also, since this study was hospital-based, the findings may not be representative of the general population.

## 6. Conclusion

Even though some predictive tests for preterm births are used routinely in developed countries, they still have poor sensitivities and positive predictive values and are very expensive, while blood measurement for magnesium concentrations is relatively cheap. Moreover, since we can deduce from this study that low serum magnesium (hypomagnesaemia) is associated with preterm onset of labour, we can then suggest that maternal hypomagnesaemia may be used as a predictor of preterm labour.

Further studies are however required to find out the other aetiologies of irritability of uterus due to low level of serum magnesium, to evaluate the role of magnesium in preterm labour and the probability of use of low serum magnesium as a marker or predictor of idiopathic group of preterm labour. This can then form the basis for formulating a proposition in the future for the use of prophylactic oral magnesium supplementation (not parenteral magnesium sulphate) among patients with higher risk for development of preterm labour.

## Figures and Tables

**Table 1:** Oyedeji socioeconomic classification scheme.

Class		Occupation
I	Upper	Professional
II		Managerial or technical

III	Middle	Skilled

IV		Partially skilled
V	Lower	Unskilled

**Table 2:** Demographic characteristics of respondents.

Characteristics	Study patients		*P*-value
	Case (preterm labour)	Control (term labour)	
	*N* (%)	*N* (%)	
Age			
16–20	7 (7.0)	13 (13.0)	0.098
21–25	15 (15.0)	17 (17.0)	
26–30	33 (33.0)	30 (30.0)	
31–35	27 (27.0)	25 (25.0)	
36–40	18 (18.0)	15 (15.0)	
Mean ± SD (years)	29.75 ± 5.24	28.63 ± 5.92	
	**Age range = 16–39 years**	
Parity			
Primigravidae	17 (17.0)	22 (22.0)	0.534
Multigravidae	83 (83.0)	78 (78.0)	
Marital status			
Single	20 (20.0)	19 (19.0)	0.064
Married	80 (80.0)	81 (81.0)	
Tribe			
Hausa	13 (13.0)	10 (10.0)	0.830
Ibo	25 (25.0)	23 (23.0)	
Yoruba	47 (47.0)	51 (51.0)	
Others	15 (15.0)	16 (16.0)	
Religion			
Christianity	62 (62.0)	53 (53.0)	0.106
Islam	33 (33.0)	46 (46.0)	
Others	5 (5.0)	1 (1.0)	
Social class			
Upper	17 (17.0)	14 (14.0)	0.139
Middle	50 (50.0)	57 (57.0)	
Lower	33 (33.0)	29 (29.0)	
Total	**100 (100.0)**	**100 (100.0)**	

**Table 3:** Relationship between serum magnesium and preterm labour.

Serum magnesium	Study patients		Total *N* (%)
	Case (preterm labour)	Control (term labour)	
	*N* (%)	*N* (%)	
<1.6 mg/dL	47 (47.0)	25 (25.0)	72 (36.0)
≥1.6 mg/dL	53 (53.0)	75 (75.0)	128 (64.0)
Total	**100 (100.0)**	**100 (100.0)**	**200 (100.0)**

Mean ± SD (mg/dL)	1.73 ± 0.4	1.93 ± 0.4	1.83 ± 0.4

Relative risk = 1.83 (95% CI—0.72–0.38); *P* value = 0.024.

**Table 4:** Comparison of the previous studies on serum magnesium level in preterm labour with the current study [14, 26–30].

Reference	Serum magnesium level	*P* value
	(mean ± SD) mg/dL	
Kurzal [26]	1.60 ± 0.46	<0.0005
Pushpo and Jagdish [27]	1.67 ± 0.23	<0.001
Smolarczyk et al. [29]	1.64 ± 0.07	<0.003
Wójcicka-Jagodzińska et al. [30]	1.63 ± 0.053	<0.001
Begum et al. [28]	1.77 ± 0.36	<0.001
Shahid et al. [14]	1.87 ± 0.3	<0.001
Present study	1.73 ± 0.4	<0.05

**Table 5:** Relationship between serum magnesium and gestational age for women with preterm delivery.

G.A. at delivery (weeks)	Serum magnesium levels		*P* value
	<1.6 mg/dL	≥1.6 mg/dL	
	*N* (%)	*N* (%)	
28-29	1 (2.1)	3 (5.7)	0.098
30-31	3 (6.4)	4 (7.5)	
32-33	6 (12.8)	8 (15.1)	
34-35	12 (25.5)	12 (22.6)	
36-<37	25 (53.2)	26 (49.1)	
Total	**47 (100.0)**	**53 (100.0)**	

**Table 6:** Relationship between serum magnesium and the major risk factors for preterm labour.

Risk factors	*N* = 200	Total serum magnesium level (mg/dL)			*P* value
		Mean ± SD (mg/dL)	Min	Max	
Maternal age					
16–20	20	1.77 ± 0.3	1.46	3.10	0.099
21–25	32	1.81 ± 0.4	1.39	2.97	
26–30	63	1.69 ± 0.3	1.30	3.00	
31–35	52	2.10 ± 0.1	1.75	2.89	
36–40	33	1.97 ± 0.2	1.66	2.45	
Social class					
Upper	31	2.01 ± 0.6	1.69	3.11	0.101
Middle	107	1.68 ± 0.5	1.30	2.97	
Lower	62	1.88 ± 0.4	1.24	2.99	
History of midtrimester miscarriage					
Yes	69	1.88 ± 0.4	1.30	2.80	0.716
No	131	1.80 ± 0.4	1.25	2.99	
History of preterm labour					
Yes	38	1.66 ± 0.5	1.30	2.60	0.059
No	162	1.87 ± 0.4	1.25	2.99	

## References

[1] March of Dimes Healthy babies, healthy business. http://www.marchofdimes.com/hbhb/.

[2] March of Dimes Premature birth. http://www.marchofdimes.com/prematurity/21191.asp.

[3] Kierse M. J. N. C. (1995). New perspectives for the effective treatment of preterm labour. *The American Journal of Obstetrics and Gynaecology*.

[4] Goldenberg R. L., Culhane J. F., Iams J. D., Romero R. (2008). Epidemiology and causes of preterm birth. *The Lancet*.

[5] Arius F. (1993). Preterm labour. *Practical Guide to High Risk Pregnancy and Delivery*.

[6] Institute of Medicine (2006). *Preterm Birth: Causes, Consequences, and Prevention*.

[7] Hofman P. L., Regan F., Jackson W. E. (2004). Premature birth and later insulin resistance. *New England Journal of Medicine*.

[8] Moutquin J. M., Cabrol D., Fisk N. M., MacLennan A. H., Maršál K., Rabinovici J. (2001). Effectiveness and safety of the oxytocin antagonist atosiban versus beta-adrenergic agonists in the treatment of preterm labour. *British Journal of Obstetrics and Gynaecology*.

[9] Lumley J. (2003). Defining the problem: the epidemiology of preterm birth. *International Journal of Obstetrics and Gynaecology*.

[10] Peacock J. L., Bland J. M., Anderson H. R. (1995). Preterm delivery: effects of socioeconomic factors, psychological stress, smoking, alcohol, and caffeine. *The British Medical Journal*.

[11] Martin R. W., Perry K. G., Hess W., Martin J. N., Morrison J. C. (1992). Oral magnesium and the prevention of preterm labor in a high-risk group of patients. *The American Journal of Obstetrics and Gynecology*.

[12] Crane J. M. G., Armson B. A., Dodds L., Feinberg R. F., Kennedy W., Kirkland S. A. (1999). Risk scoring, fetal fibronectin, and bacterial vaginosis to predict preterm delivery. *Obstetrics and Gynecology*.

[13] Goffinet F. (2005). Primary predictors of preterm labour. *International Journal of Obstetrics and Gynaecology*.

[14] Shahid A. R., Hosna A. U., Tahmina H. Z. (2010). Hypomagnesaemia in pregnancy: a predictor of preterm labour. *Journal of Dhaka Medical College*.

[15] Kamal S., Sharan A., Kumar U., Shahi S. K. (2003). Serum magnesium level in preterm labour. *Indian Journal of Pathology and Microbiology*.

[16] Elliot J. P. (1983). Magnesium sulfate as a tocolytic agent. *The American Journal of Obstetrics and Gynecology*.

[17] Watras J. (1985). Effects of Mg^2+^ on calcium accumulation by two fractions of sarcoplasmic reticulum from rabbit skeletal muscle. *Biochimica et Biophysica Acta: Biomembranes*.

[18] Spisso K. R., Harbert G. M., Thiagarajah S. (1982). The use of magnesium sulfate as the primary tocolytic agent to prevent premature delivery. *The American Journal of Obstetrics and Gynecology*.

[19] Begum A. A., Das T. R. (2010). Low serum magnesium in preterm labour. *Journal of Bangladesh College of Physicians and Surgeons*.

[20] Grimes D. A., Nanda K. (2006). Magnesium sulfate tocolysis: time to quit. *Obstetrics and Gynecology*.

[21] Schlesselman J. J. (1974). Sample size requirements in cohort and case-control studies of disease. *The American Journal of Epidemiology*.

[22] Oyedeji G. A. (1985). Socioeconomic status and cultural background of hospitalized children in Ilesa. *Nigerian Journal of Paediatrics*.

[23] Magnesium colorimetric method.

[24] Tietz N. (1986). *Textbook of Clinical Chemistry*.

[25] (2008). *The SPSS System for Windows [Computer Program] Version 17*.

[26] Kurzal R. B. (1991). Serum magnesium level in pregnancy and in preterm labour. *The American Journal of Perinatology*.

[27] Pushpo D., Jagdish W. M. A. (1991). A study of serum magnesium level in preterm labour. *Journal of Obstetrics and Gynaecology of India*.

[28] Begum H., Shamsuddin L., Khatun S. (2004). Relationship of preterm labour with serum magnesium level. *Bangladesh Journal of Obstetrics & Gynaecology*.

[29] Smolarczyk R., Wójcicka-Jagodzińska J., Romejko E., Piekarski P., Czajkowski K., Teliga J. (1997). Calcium-phosphorus-magnesium homeostasis in women with threatened preterm delivery. *International Journal of Gynecology and Obstetrics*.

[30] Wójcicka-Jagodzińska J., Romejko E., Piekarski P., Czajkowski K., Smolarczyk R., Lipiński T. (1998). Second trimester calcium-phosphorus-magnesium homeostasis in women with threatened preterm delivery. *International Journal of Gynecology and Obstetrics*.

[31] Arikan G. M., Panzitt T., Gücer F. (1999). Course of maternal serum magnesium levels in low-risk gestations and in preterm labor and delivery. *Fetal Diagnosis and Therapy*.

[32] Han S., Crowther C. A., Moore V. (2010). Magnesium maintenance therapy for preventing preterm birth after threatened preterm labour. *Cochrane Database of Systematic Reviews*.

[33] Crowther C. A., Hiller J. E., Doyle L. W. (2002). Magnesium sulphate for preventing preterm birth in threatened preterm labour. *Cochrane Database of Systematic Reviews*.

[34] Cunningham F., Leveno K., Bloom S., Hauth J., Gilstrap L., Wenstrom K. (2005). *Williams Obstetrics*.

[35] Kamal S., Sharan A., Kumar U., Shahi S. K. (2003). Serum magnesium level in preterm labour. *Indian Journal of Pathology and Microbiology*.

[36] Carr-Hill R. A., Hall M. H. (1985). The repetition of spontaneous preterm labour. *British Journal of Obstetrics and Gynaecology*.

